# A universally conserved OMV-YidR conjugate nanovaccine confers broad protection against hypervirulent and carbapenem-resistant *Klebsiella pneumoniae*

**DOI:** 10.1080/22221751.2026.2731500

**Published:** 2026-09-07

**Authors:** Shuangjun He, Yang Zhang, Ahui Song, Chenyu Fan, Feng Zhao, Wei Zhou, Cuiying Xie, Chu Guo, Shengliang Chen, Yi Chen

**Affiliations:** a Department of Emergency, Shanghai Jiao Tong University School of Medicine, Renji Hospital, Shanghai, People’s Republic of China; b Pujiang Hospital, Shanghai, People’s Republic of China; c Division of Gastroenterology and Hepatology, Key Laboratory of Gastroenterology and Hepatology, Shanghai Institute of Digestive Disease, Shanghai Jiao Tong University School of Medicine, Renji Hospital, Shanghai, People’s Republic of China

**Keywords:** *Klebsiella pneumoniae*, antibiotic resistance, nanovaccine, outer membrane vesicle, YidR protein

## Abstract

The escalating crisis of multi-drug resistant (MDR) *Klebsiella pneumoniae* (KP) renders standard antibiotic treatments increasingly ineffective, necessitating a shift towards alternative approaches like vaccination. However, the development of a broadly protective vaccine remains hindered by the pathogen's extensive serotype diversity. To overcome this limitation, we targeted YidR – a highly conserved protein with potential as a universal antigen – and engineered a semi-synthetic conjugate vaccine by covalently linking it to KP-derived outer membrane vesicles (OMVs). The OMV-YidR conjugate successfully overcame the poor intrinsic immunogenicity of soluble YidR, eliciting robust antigen-specific IgG titres >20,000-fold higher than the unconjugated protein. Furthermore, the conjugate vaccine drove a shift from a weak Th2 bias to a potent, protective Th1/Th17 cellular profile. Crucially, this enhanced immunogenicity translated into broad-spectrum efficacy: while unconjugated OMVs protected only against the homologous strain, the OMV-YidR conjugate conferred complete (100%) survival against lethal challenges with diverse clinical isolates, including hypervirulent and carbapenem-resistant lineages. Mechanistic and comparative analyses revealed that covalent linkage – rather than simple physical co-administration – was essential for this superior efficacy, which was correlated with the preferential trafficking and uptake of the antigen by B cells in the draining lymph nodes. These findings establish the OMV-YidR conjugate as a highly potent, universal vaccine candidate capable of bypassing serotype limitations to prevent untreatable MDR *K. pneumoniae* infections.

## Introduction

*Klebsiella pneumoniae* (KP) has emerged as a critical pathogen within the “ESKAPE” group, accounting for a substantial proportion of hospital-acquired infections, including pneumonia, sepsis, and urinary tract infections [1]. The emergence of multidrug-resistant (MDR) strains, particularly carbapenem-resistant KP (CRKP), has limited therapeutic options and is associated with high mortality rates [2, 3]. Furthermore, the convergence of MDR and hypervirulence has led to the rise of carbapenem-resistant hypervirulent KP (CR-hvKP), which can cause severe, metastatic infections in healthy populations and represents a dire clinical challenge [2].

Vaccination offers a cost-effective alternative to combat KP infections, yet there are currently no licenced vaccines available for human use. Traditional vaccine development strategies have focused largely on surface polysaccharides, specifically the capsular polysaccharide (K antigen) and lipopolysaccharide (O antigen) [4–6]. However, the utility of these targets is severely constrained by the pathogen's extensive heterogeneity; there are at least 77 distinct capsular serotypes and multiple O-antigen serogroups, making it difficult to achieve broad-spectrum coverage with a single formulation [4, 7, 8]. Additionally, polysaccharide-based vaccines often fail to induce T-cell-dependent immune responses, which are crucial for establishing long-term immunological memory, particularly in vulnerable populations such as infants and the elderly [8].

To overcome the limitations of serotype-dependent antigens, recent research has pivoted towards conserved surface proteins that are present across diverse strains. YidR, a putative ATP/GTP-binding protein, has emerged as a highly promising candidate due to its universal conservation (>97% homology) across classical, hypervirulent, and multidrug-resistant KP clinical isolates, regardless of capsular or O-antigen serotype [9, 10]. Furthermore, YidR functions as an active virulence factor, and preclinical studies have demonstrated that recombinant YidR (rYidR) or DNA vaccine platforms can elicit protective immunity in murine sepsis models [9, 11]. However, recombinant subunit vaccines often exhibit low intrinsic immunogenicity and typically fail to confer durable, high-titer protection without the addition of potent external adjuvants.

Bacterial outer membrane vesicles (OMVs) provide an ideal platform to overcome the poor immunogenicity of conserved protein subunits [12]. Naturally secreted by Gram-negative bacteria, OMVs are spherical, bilayered nanostructures (20–200 nm) that encapsulate periplasmic contents, outer membrane proteins, and intrinsic pathogen-associated molecular patterns (PAMPs), most notably lipopolysaccharide (LPS) [13]. Due to their particulate nature and the presence of intrinsic immunostimulatory molecules, OMVs can be efficiently taken up by antigen-presenting cells (APCs) and induce both humoral and cell-mediated immunity [13, 14]. Furthermore, OMVs can be engineered to display heterologous antigens, thereby enhancing the immunogenicity of the carried cargo [14, 15].

While OMVs can be engineered to carry heterologous antigens, simply co-administering OMVs with soluble proteins often fails to maximize synergistic immunity [16, 17]. In this study, we established a therapeutic and mechanistic proof-of-concept for this technology by covalently linking YidR, serving as a representative, conserved and biologically validated model antigen, directly to the surface of KP-derived OMVs. We hypothesized that this stable physical linkage would overcome the limitations of soluble protein immunization by forcing the co-delivery of the conserved antigen and the OMV's intrinsic PAMPs to the same immune cells. We evaluated the immunological superiority of this OMV-YidR conjugate compared to standard protein and physical mixtures, investigated its unique cellular trafficking mechanisms within draining lymph nodes, and assessed its capacity to confer broad-spectrum survival against lethal challenges with diverse, heterologous KP strains, including untreatable hypervirulent and carbapenem-resistant lineages. This approach represents a promising strategy for developing a broadly protective, serotype-independent vaccine against multidrug-resistant KP.

## Materials and methods

### Bacteria culture and OMV isolation

Four KP strains, ATCC 4208, ATCC 43816, BAA-1705 and BAA-2342, were all purchased from ATCC and cultured according to ATCC’s protocols. In brief, BAA-1705 was cultured in ATCC Medium 18 while the other 3 strains were cultured in ATCC Medium 3. All cultures were maintained at 37°C, 200 rpm.

ATCC 4208 was used to produce OMVs in the current study and OMVs were isolated as previously described with modifications [18]. In brief, cultured KP was first pelleted, and supernatant was filtered through a 0.22 μm filter to remove bacteria and debris, then the cleared culture supernatant was concentrated using 100 kD Amicon ultrafiltration tubes (Merck). The concentrated supernatant was filtered through a 0.22 μm filter to remove potential precipitations before OMVs were purified with qEV 35 nm size exclusion columns (IZON), according to the manufacturer’s instructions. Purified OMVs were concentrated with 100 kD Amicon ultrafiltration tubes, 0.22 μm filtered, aliquoted and stored at −80°C till use.

### Protein expression and purification

**Figure 1.**
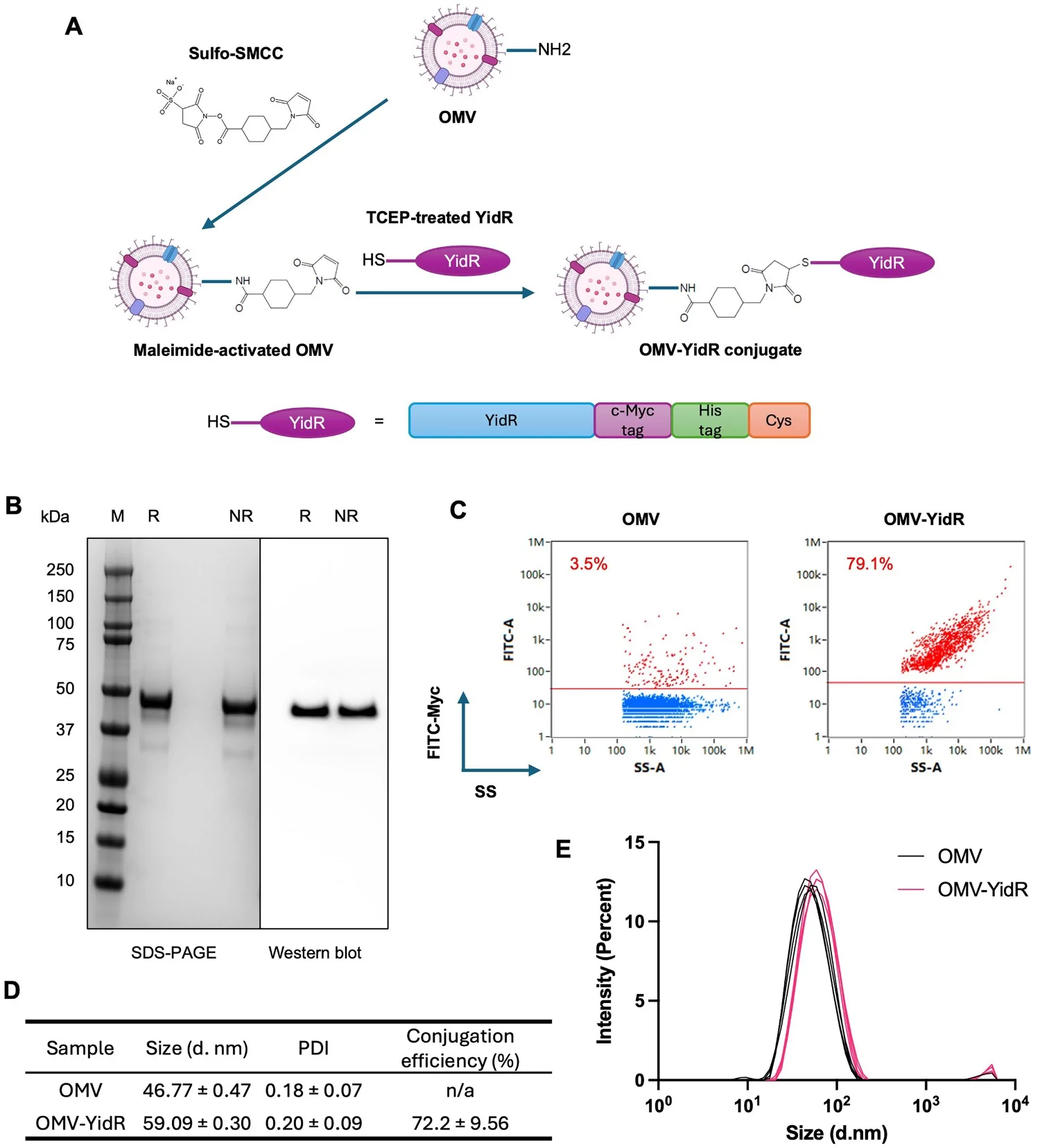
Characterization of the OMV-YidR nanovaccine conjugate. (A) OMV-YidR conjugation strategy. YidR protein was conjugated onto OMV surface using click chemistry with Sulfo-SMCC heterobifunctional crosslinkers following a 2-step conjugation strategy. OMVs were first conjugated to Sulfo-SMCC through NHS-NH2 conjugation, and then YidR protein, after TCEP treatment, was added onto through thiol-maleimide reaction. (B) Purified YidR protein was characterized by SDS-PAGE and western blot under reduced (R) and non-reduced (NR) conditions. (C) The conjugation efficiency of YidR onto OMV was determined by Nano-FCM. One representative data is shown. (D) The size, PDI and conjugation efficiency of OMVs before and after YidR conjugation. (E) Representative DLS profiles of OMV and OMV-YidR.

YidR protein was expressed in *E. coli* BL21 (DE3) strain, as previously described with modifications [9]. DNA sequence coding for YidR protein was retrieved from GenBank (Accession number: NZ_KN046818.1), and a c-Myc tag (for detection), 8x His tag (for purification), and a cysteine (for conjugation) were added to the C-terminus (Figure 1(A)). The final sequence was synthesized and cloned into the pET28a expression vector by GenScript. pET28a-YidR plasmid was then transformed into *E. coli* BL21 (DE3) and cultured in LB broth at 37 °C, 200 rpm, until the OD600 reached around 0.5–0.6. Protein expression was then induced with the addition of 1 mM IPTG (Merck) followed by culturing at 25 °C, 200 rpm, for another 5 h. Cells were subsequently harvested and washed with DPBS and resuspended in DPBS supplemented with protease inhibitor cocktail (Proteintech), and sonicated on ice with a probe sonicator (HUXI) for 5 repeats of 30 s sonication and 60 s pause. Sonicated cells were centrifug ed at 12,000 g for 30 min at 4 °C and the resulting supernatant was loaded onto an HisTrap excel column (Cytiva) to purify the YidR protein. The eluted fractions with target protein were concentrated with a 10 kD Amicon ultrafiltration tube and further purified by size exclusion chromatography (SEC) with a Superdex 200 Increase 10/300 GL column (Cytiva) on an ÄKTA Pure system (Cytiva). Purified protein was finally aliquoted and stored at −80 °C till use.

### SDS-PAGE and western blot

Protein samples were first mixed with LDS sample loading buffer with or without reducing reagent (Thermo Fisher Scientific), and then separated by a 4–12% Bis-Tris gel (Yeasen). For protein purity check, the gel was stained with Commassie Blue Fast Stain Solution (Yeasen), washed with water, and imaged by a gel imaging system (Bio-Rad). For Western blot analysis, gel-separated proteins were transferred onto a 0.45 μm PVDF membrane. The membrane was then blocked with 5% non-fat milk, and incubated with HRP-conjugated mouse anti-c-Myc tag antibody (HRP-60003; Proteintech). After extensive washes with PBST, the immuno-bands were detected with Enhanced ECL Chemiluminescent Substrate Kit (Yeasen).

### Alexa Fluor® 488 labelling of YidR protein

Alexa Fluor® 488 Conjugation Kit – Lightning-Link® (ab236553; Abcam) was used in the current study for conjugation. In brief, purified YidR protein in PBS was diluted to 1 mg/ml, and then the conjugation reaction was set up according to the manufacturer’s instructions. After 1 h incubation at room temperature in the dark, the reaction was stopped by the addition of the Quencher reagent. Conjugated YidR-AF488 was purified by SEC with a Superdex 200 Increase 10/300 GL column (Cytiva).

### OMV-YidR conjugation and purification

YidR protein was conjugated onto OMV surface using click chemistry with Sulfo-SMCC heterobifunctional crosslinkers (Thermo Fisher Scientific), according to the manufacturer’s instructions with modifications. In brief, purified OMVs (2.5 mg/ml) were first incubated with Sulfo-SMCC linker (25 mg/ml) for 30 min at room temperature followed by SEC purification with qEV 35 nm columns (IZON) to produce maleimide-activated OMVs. Then, the maleimide-OMVs (0.25 mg/ml) were incubated with TCEP-reduced YidR protein (0.25 mg/ml) for 1 h at room temperature and overnight at 4°C to conjugate YidR protein onto OMV surface. Following conjugation, the conjugated material was incubated with 50 mM L-cysteine for 1 h at room temperature to quench unreacted maleimide group on OMV surface. Afterwards, conjugate was purified by SEC with qEV 35 nm columns (IZON), concentrated with 100 kD Amicon ultrafiltration columns (Merck), filtered through 0.22 μm filters, aliquoted and stored at −80 °C till use.

### Dynamic light scattering (DLS)

The hydrodynamic diameter and polydispersity index (PDI) of OMVs were characterized by Dynamic Light Scattering (DLS) using a Zetasizer Ultra (Malvern Panalytical), according to the manufacturer’s instructions. In brief, samples were equilibrated at 25°C in PBS and measured using a 633 nm laser with Non-Invasive Backscatter (NIBS) detection. Data were processed using ZS Xplorer software.

### NanoFCM

NanoFCM was used to assess the conjugation efficiency of YidR protein onto OMV surface. Unconjugated OMV or conjugated OMV-YidR was first diluted to 2 × 10^10^ particles/ml with 0.22 μm filtered PBS, and then incubated with CoraLite® Plus 488-conjugated mouse anti-c-Myc tag antibody (CL488-60003; Proteintech) for 1 h at room temperature in the dark. Following incubation, the stained samples were diluted 100 folds with 0.22 μm filtered PBS and then evaluated on NanoFCM.

### Mouse vaccination and sampling

Six- to eight-week-old, SPF female C57BL/6J mice (Shanghai Lingchang Bio-Technology Co. Ltd) were used in the current project for all *in vivo* tests. All protocols involving animals were reviewed and approved by the local ethical review board (Approval No. IACUC-2025-NI-058). Mice were intramuscularly (i.m.) immunized twice at 4-week intervals, with either PBS, YidR protein (1 μg), OMV (1 μg), or OMV-YidR (1 μg) in 50 μl PBS. Blood samples were taken at Week 0, 4, and 6 for antigen-specific antibody measurements, and spleens were taken at Week 6 to measure antigen-specific cellular responses. The Week 0 serum samples served as a pre-immunization screening to confirm the absence of pre-existing immunity or abnormal background signal against YidR or OMV antigens in all study animals prior to treatment allocation.

### Bacteria challenge study

To identify the lethal dose for the KP strains used in the current study, naïve C57BL/6J mice were challenged intraperitoneally (i.p.) with various doses of KPs in 100 μl PBS, and then survival rate was monitored every 12 h for the first 3 days, followed by daily until Day 7. The lowest dose that resulted in 100% animal death was chosen as the lethal dose of each strain.

To determine the protective effect of the vaccine formulations, C57BL/6J mice were first received two vaccinations (i.m.) according to the schedule illustrated in Figure 3(A). Three weeks after the second vaccination, mice were challenged i.p. with lethal doses of different KP strains, and animal survival rate was recorded for 7 days.

To evaluate the impact of vaccination on systemic bacterial distribution and organ clearance, C57BL/6J mice were first received two vaccinations (i.m.) according to the schedule illustrated in Figure 6(A). Three weeks after the second vaccination, mice were challenged i.p. with non-lethal doses of different KP strains. At 48 h post challenge, animals were sacrificed and the lungs, livers, and spleens were harvested, weighed, and homogenized in sterile PBS. The tissue homogenates were serially diluted and plated on LB agar to determine the viable bacterial load, expressed as colony-forming units per gram of tissue (CFU/g).

### ELISA

YidR-specific and OMV-specific IgG titres were measured by direct ELISA, as previously described with modifications [14, 19]. In brief, MaxiSorp ELISA plates (Thermo Fisher Scientific) were coated with 1 μg/ml YidR protein (for YidR-specific IgG) or OMV (OMV-specific IgG) in PBS overnight at 4 °C. To generate IgG standard curve, each plate contained wells coated with anti-mouse Kappa and Lamda chains (Proteintech). After coating, plates were washed and blocked with 1% BSA, followed by incubation of serially diluted serum samples or standard mouse IgG (for standard curve). After further washes, plates were then incubated with HRP-conjugated anti-mouse IgG (Proteintech). Following incubation, plates were washed again and colorimetric reaction was developed with TMB substrate (Biotime) and stopped with Stop solution (Biotime). Plates were then read by a spectrophotometer (Thermo Fisher Scientific) at OD450 (testing wavelength) and OD800 (reference wavelength). Antibody titres were calculated from the standard curve generated on each plate.

### Cytokine measurement

T cell-associated immune response was assessed by measuring antigen-specific Th-related cytokine expression from antigen-restimulated splenocytes, as previously described with modifications [14]. In brief, 2 weeks after the second vaccination, mice were sacrificed and spleens were harvested. Splenocytes were isolated with Mouse Lymphocyte Separation Medium (DAKEWE), according to the manufacturer’s instructions. Isolated splenocytes were then cultured in RPMI 1640 medium (HyClone, Cytiva) supplemented with 10% FBS (HyClone, Cytiva), in the presence or absence of stimulation. The following stimulations were used: 10 μg/ml YidR protein (antigen-specific restimulation), 5 μg/ml Con A (positive control stimulation) and PBS (protein solvent; negative control stimulation). The cells were cultured for 7 days and then supernatants were harvested and cytokines from stimulated cells were quantified by Luminex or ELISA.

For Luminex quantification, a Th1/Th2/Th9/Th17/Th22/Treg Cytokine 17-Plex Mouse ProcartaPlex™ Panel (Thermo Fisher Scientific) was used, and evaluation was performed on a BioPlex 200 (Bio-Rad) platform, according to the manufacturers’ instructions.

For ELISA quantification, ELISA kits for mouse IL-4 (KE10010; Proteintech) and IFN-γ (ab100689; Abcam) were used. All procedures were performed strictly according to the manufacturers’ instructions.

### Opsonophagocytic killing (OPK) assay

The neutrophil-mediated killing was assessed by OPK assay as previously described with modifications [5, 20, 21]. In brief, mid-log phase bacteria were washed in 1x PBS and opsonized with heat-inactivated immunized mouse sera (56°C for 30 min) in RPMI-1640 (Gibco) containing 10% LB for 30 min at 37°C in 96-well plates on an orbital shaker. Following this initial opsonization, freshly isolated human neutrophils and exogenous baby rabbit complement were added to the wells. To facilitate cell-bacteria contact, plates were incubated on an orbital shaker for 1 hour at 37°C with 5% CO_2_. Finally, well contents were mixed and the neutrophils were lysed in H_2_O to release intracellular bacteria. The resulting lysates were serially diluted in 1x PBS and plated on LB agar plates for viable CFU enumeration. Bacterial survival was normalized against control wells containing neutrophils, complement, and heat-inactivated serum from PBS-mock immunized mice.

### Flow cytometry

Antigen uptake in the draining lymph nodes (dLNs) was measured by flow cytometry, as previously described with modifications [14]. In brief, C57BL/6J mice were first dosed i.m. with 10 μg YidR-AF488 or OMV-YidR-AF488, and 1 h post-injection, mice were sacrificed and dLNs were harvested. Single cell suspension was generated by pressing the dLNs through a 70 μm cell strainer (BD Falcon). Isolated cells were then washed and resuspended in DPBS and stained with LIVE/DEAD™ Fixable Aqua Dead Cell Stain Kit (Thermo Fisher Scientific), following the manufacturer’s instructions. Following live/dead staining, cells were washed and resuspended in FACS staining buffer (Proteintech), and then sequentially incubated with mouse Fc block (BioLegend) and staining antibodies. The following antibodies were used in the current study, and they were all purchased from BioLegend: BV650-Ly-6G (neutrophil), APC/CY7-CD49b (NK), BV711-CD3e (T cell), PE-CD11b (monocyte), BV785-CD11c (DC), BV605-CD19 (B cell), and BV421-F4/80 (macrophage). After staining, cells were washed and evaluated on a BD LSRFortessa flow cytometer. Data were analyzed using FlowJo 10.8.1.

### Statistical analysis

Statistical analyses were conducted using GraphPad Prism version 10. All results are presented as the mean ± standard deviation (SD), with statistical significance defined as a *p*-value less than 0.05. For analyses involving two groups, the Mann-Whitney test was applied. In instances where three or more groups were compared, the Kruskal-Wallis test was utilized, followed by Dunn’s test for multiple comparisons.

## Results

### Preparation and characterization of OMV-YidR conjugate

The heterobifunctional crosslinker Sulfo-SMCC was utilized to covalently couple purified YidR protein to the surface of KP-derived OMVs. Sulfo-SMCC was selected for its ability to form stable, non-cleavable thioether bonds, ensuring antigen retention on the carrier *in vivo*. The conjugation was achieved through a two-step mechanism: the N-hydroxysuccinimide (NHS) ester group reacted with primary amines on the OMV surface proteins, followed by the reaction of the maleimide group with sulfhydryl groups on the antigen. To facilitate this conjugation, the recombinant YidR construct was engineered with a C-terminal cysteine residue to provide the necessary free sulfhydryl group, alongside c-Myc and His-tags to aid in detection and purification, respectively (Figure 1(A, B)).

The resulting OMV-YidR conjugate was characterized using Nano-Flow Cytometry (Nano-FCM) and Dynamic Light Scattering (DLS). Nano-FCM analysis demonstrated a high conjugation efficiency, with 72.2% ± 9.56% of the OMV population successfully displaying YidR on the surface (Figure 1(C, D)). DLS analysis indicated a slight increase in hydrodynamic diameter for the OMV-YidR conjugate compared to unconjugated OMVs, indicating successful surface protein loading. Importantly, the Polydispersity Index (PDI) remained stable throughout the process, indicating that the vesicles maintained their structural integrity with minimal aggregation (Figure 1(D, E)). Together, these data confirm the successful generation of a stable, high-density OMV-YidR conjugate.

### Conjugation of YidR protein to the OMV surface significantly enhances YidR-specific humoral and cellular responses in mice

**Figure 2.**
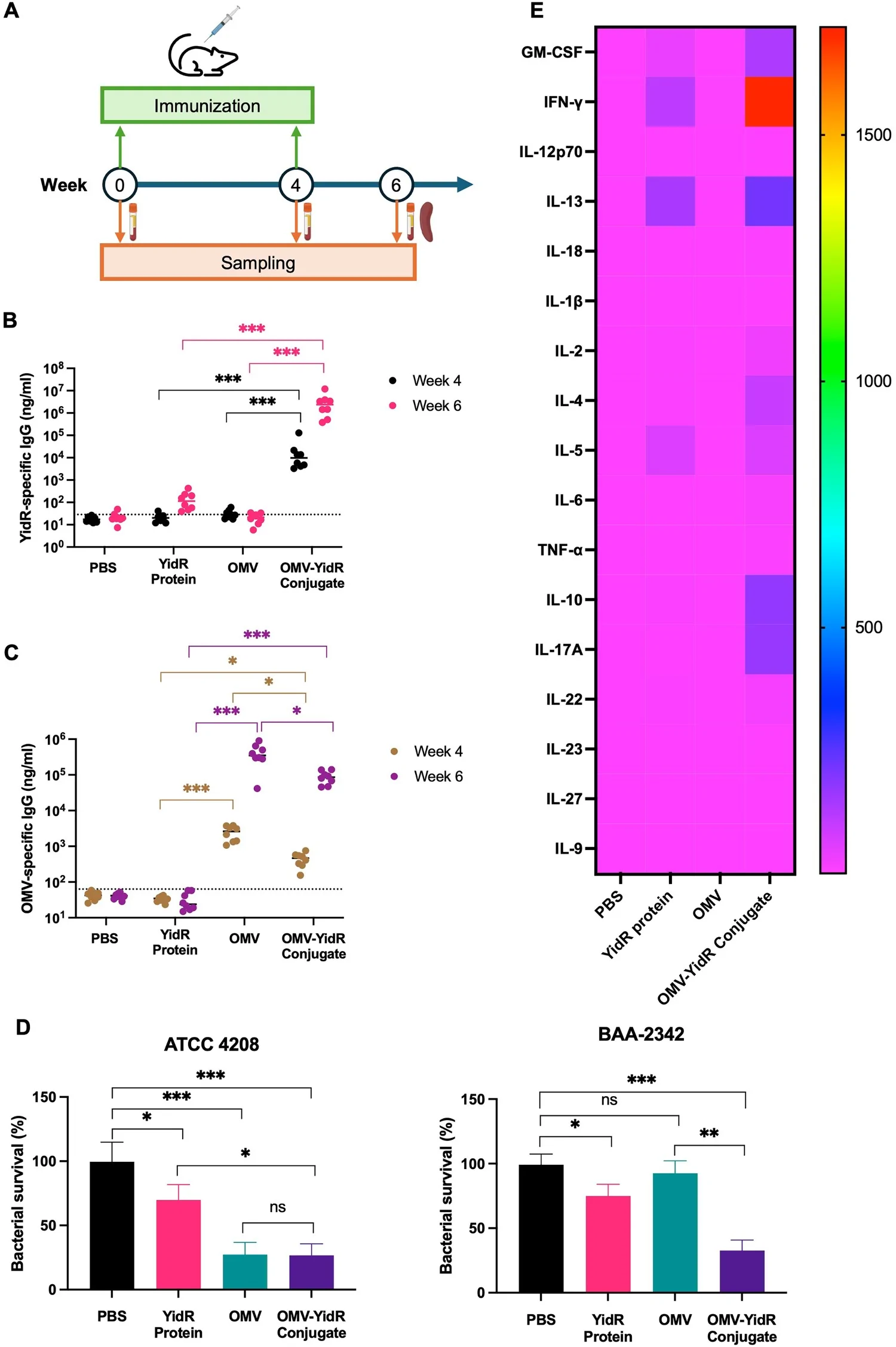
OMV-YidR conjugate elicits enhanced humoral and cellular responses against YidR. (A) Immunization strategy. C57BL/6J mice were first immunized twice at 4-week intervals and then serum samples were collected at week 0, 4, and 6 for antibody measurement and spleens were harvested at week 6 for T-cell mediated response evaluation. (B) YidR-specific and (C) OMV-specific IgG were measured in serum samples at week 4 and 6 by ELISA (*n* =   8). *, *p* <   0.05; **, *p* <   0.01; ***, *p* <   0.001. (D) The OPK activity of immunized sera at Week 6 against homologous ATCC 4208 and heterologous BAA-2342 strains. ns, statistically not significant; *, *p* < 0.05; **, *p* < 0.01; ***, *p* < 0.001. (E) Splenocytes were isolated at week 6 and restimulated ex vivo with purified YidR protein, and secreted Th1/2/9/17/22/Treg-related cytokines were measured by Luminex (*n* = 8). Heat map shows the fold change of individual cytokine expression relative to the control group (PBS group).

To determine if physical conjugation of YidR to the OMV delivery vehicle could potentiate immune responses compared to soluble protein, C57BL/6J mice were immunized intramuscularly on day 0 and day 28, with either PBS or 1 μg of either soluble YidR, unconjugated OMVs, or the OMV-YidR conjugate (Figure 2(A)).

YidR-specific antibody responses were quantified by ELISA. Soluble YidR protein elicited a negligible humoral response, with YidR-specific IgG barely detectable at Week 4 and remained at a very low level at Week 6. In contrast, the OMV-YidR conjugate induced robust seroconversion. YidR-specific IgG titres in the conjugate group were significantly elevated, showing a 1,145-fold increase at Week 4 and a 21,544-fold increase at Week 6 compared to the soluble protein group (Figure 2(B)). As expected, no YidR-specific antibodies were detected in the unconjugated OMV control group. Responses against the OMV carrier were also assessed; both the OMV-only and OMV-YidR groups generated strong anti-OMV IgG at Weeks 4 and 6 (Figure 2(C)). The OMV IgG titres were slightly higher in the OMV-only group, which is likely attributable to the slightly lower total mass of OMV vesicles in the conjugate dose (normalized to total protein mass) or potential steric shielding of OMV surface epitopes by the conjugated YidR antigen.

To determine if the robust IgG responses elicited by the conjugate translated into functional antimicrobial activity, we performed an opsonophagocytic killing (OPK) assay against both the OMV-production strain (ATCC 4208) and a clinically relevant carbapenem-resistant strain (BAA-2342). For the assay against the homologous ATCC 4208 strain, serum from the soluble YidR group elicited only very weak opsonophagocytic activity, whereas both the OMV and OMV-YidR conjugate groups demonstrated significantly higher and comparable bacterial killing capacity (Figure 2(D)). This indicates that against the parent strain, OMV-derived surface antigens provide sufficient targets for functional antibodies. However, a distinct functional divergence was observed against the heterologous MDR strain BAA-2342. While the YidR group maintained a similarly low level of activity – substantiating that YidR is a conserved but weakly immunogenic antigen – the OMV-only group exhibited only marginal killing activity. This result reinforces the observation that OMV-induced immunity is largely strain-specific and lacks broad cross-reactivity. In contrast, the OMV-YidR conjugate maintained high levels of potent bacterial killing against BAA-2342, confirming that the covalent conjugation of YidR to the OMV surface successfully generated a broadly effective antibody response (Figure 2(D)). These findings provide evidence that the OMV-YidR conjugate not only increases the magnitude of the antibody response but also ensures the production of highly functional opsonizing antibodies capable of empowering innate effector cells for broad-spectrum clearance.

To characterize T cell immunity, spleens were harvested at Week 6, and splenocytes were restimulated *ex vivo* with purified YidR. Cytokine production was analyzed using a 17-plex panel (Figure 2(E) and Figure S1). In the soluble YidR group, cytokine levels were generally low and skewed towards a Th2 profile, with IL-13 showing the most distinct elevation. By contrast, the OMV-YidR conjugate elicited a significantly broader and more potent cellular response. While the conjugate formulation amplified the production of nearly all measured cytokines – including GM-CSF, IFN-γ, IL-2, IL-4, IL-5, IL-10, and IL-17A – the profile exhibited a shift towards a Th1/Th17 phenotype. The most notable change was observed in IFN-γ, which showed the highest fold-increase in the panel, marking a transition away from the IL-13 dominance seen in the protein group. Additionally, substantial increases in IL-17A and IL-10 were observed compared to the protein group. These results indicate that OMV delivery does not merely enhance the magnitude of the response, but qualitatively shifts the bias towards a combined Th1/Th17 profile.

### OMV-YidR-induced immune response offers broad protection against lethal KP challenge in mice

To evaluate the protective efficacy and breadth of the vaccine candidate, a lethal challenge model was established using 4 distinct KP strains: ATCC 4208 (the OMV production strain), ATCC 43816 (a hypervirulent strain), and two clinically relevant carbapenem-resistant isolates, BAA-1705 and BAA-2342. Prior to the efficacy study, the lethal dose (LD_100_) for each strain was empirically determined by challenging naïve mice intraperitoneally (i.p.) with ascending bacterial counts, and the lowest dose resulting in 100% mortality within 7 days was selected for subsequent experiments (Figure S2).

**Figure 3.**
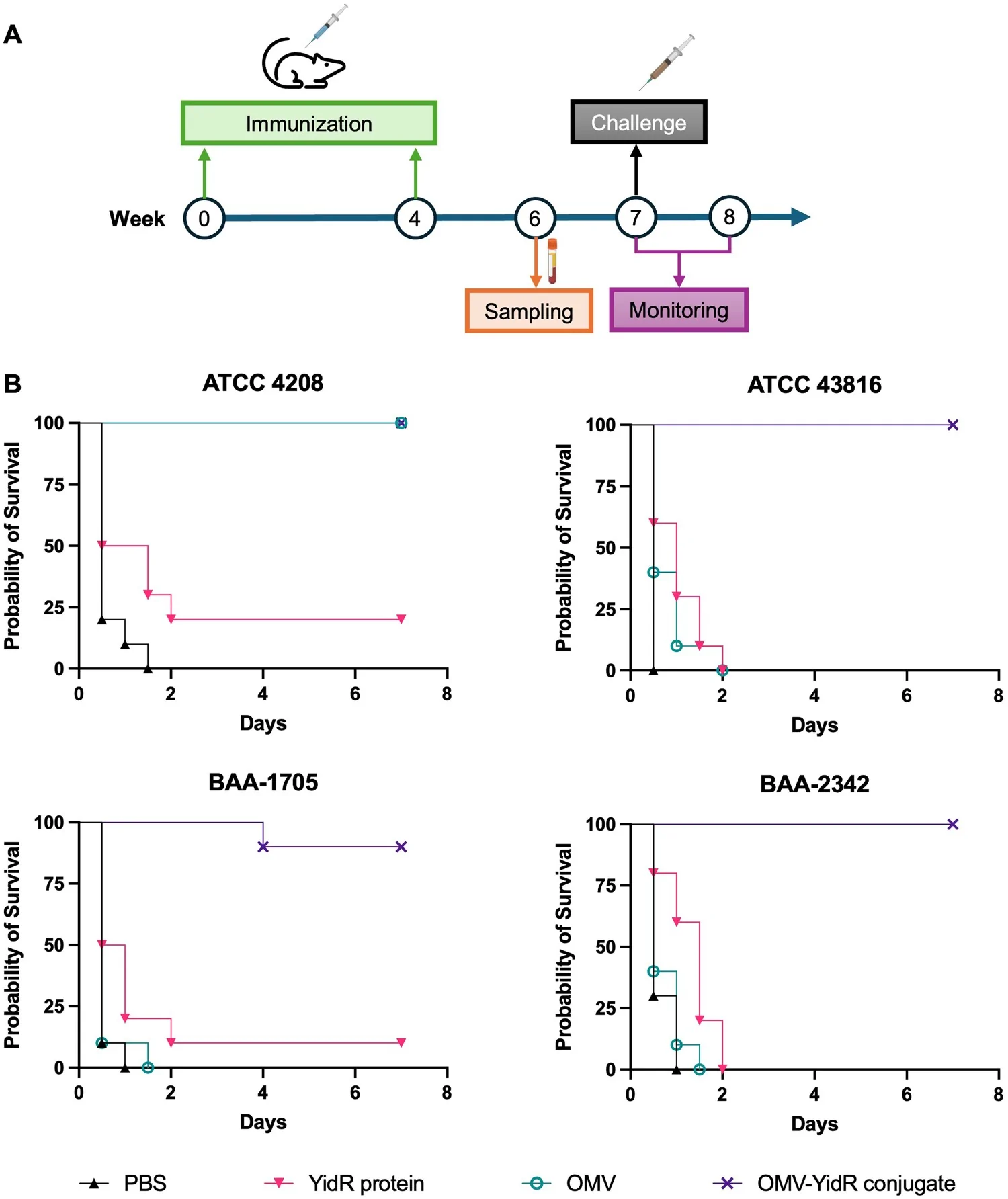
OMV-YidR conjugate induces protective immunity against homologous and heterologous KP challenge. (A) Immunization and challenge strategy. C57BL/6J mice were first immunized twice at 4-week intervals and then challenged at week 7 with a lethal dose of homologous (ATCC 4208: 5 × 10^7 CFU) and heterologous KP strains (ATCC 43816: 5 × 10^4 CFU, BAA-1705: 1 × 10^8 CFU, and BAA-2342: 1 × 10^8 CFU). (B) Animal survival was monitored for 7 days after challenge (*n* = 10).

For the protection study, C57BL/6J mice were immunized twice at a 4-week interval and subsequently challenged with the determined lethal doses. Survival was monitored for 7 days post-infection (Figure 3(A)). Protective immunity against the homologous strain, ATCC 4208, was high in both OMV and OMV-YidR groups. Mice immunized with either unconjugated OMVs or the OMV-YidR conjugate exhibited 100% survival, indicating that against the strain from which the OMVs were derived, the OMV carrier itself provides sufficient protective antigens. By contrast, the PBS control group succumbed to infection by Day 2. The soluble YidR protein conferred only marginal protection (20% survival) (Figure 3(B)).

However, a stark difference in efficacy was observed when mice were challenged with heterologous, hypervirulent, and multi-drug resistant strains. In the challenge with the hypervirulent strain ATCC 43816, the OMV-YidR conjugate provided complete protection (100% survival). In contrast, no protection was observed in the groups receiving unconjugated OMVs or soluble YidR, with 100% mortality recorded by Day 2. This failure of the unconjugated OMV group highlights that OMV-derived immunity is often strain-specific, whereas the addition of the conserved YidR antigen bridges this gap. This superior cross-protection was further demonstrated against the carbapenem-resistant isolates. For strain BAA-2342, the OMV-YidR conjugate again conferred 100% protection, while all other groups (PBS, YidR, and unconjugated OMV) experienced 100% mortality by Day 2. Similarly, against strain BAA-1705, the conjugate group maintained a 90% survival rate (with a single mortality occurring on Day 4), whereas the soluble YidR group showed only 10% survival, and the OMV and PBS groups succumbed completely by Day 2.

Collectively, these survival data demonstrate that while OMVs alone can protect against their specific parent strain, the OMV-YidR conjugate is required to generate the broad-spectrum immunity necessary to survive infection by diverse, hypervirulent, and antibiotic-resistant KP lineages.

### OMV-YidR conjugate induces a stronger YidR-specific immune response than a physical mix or standard protein adjuvant

**Figure 4.**
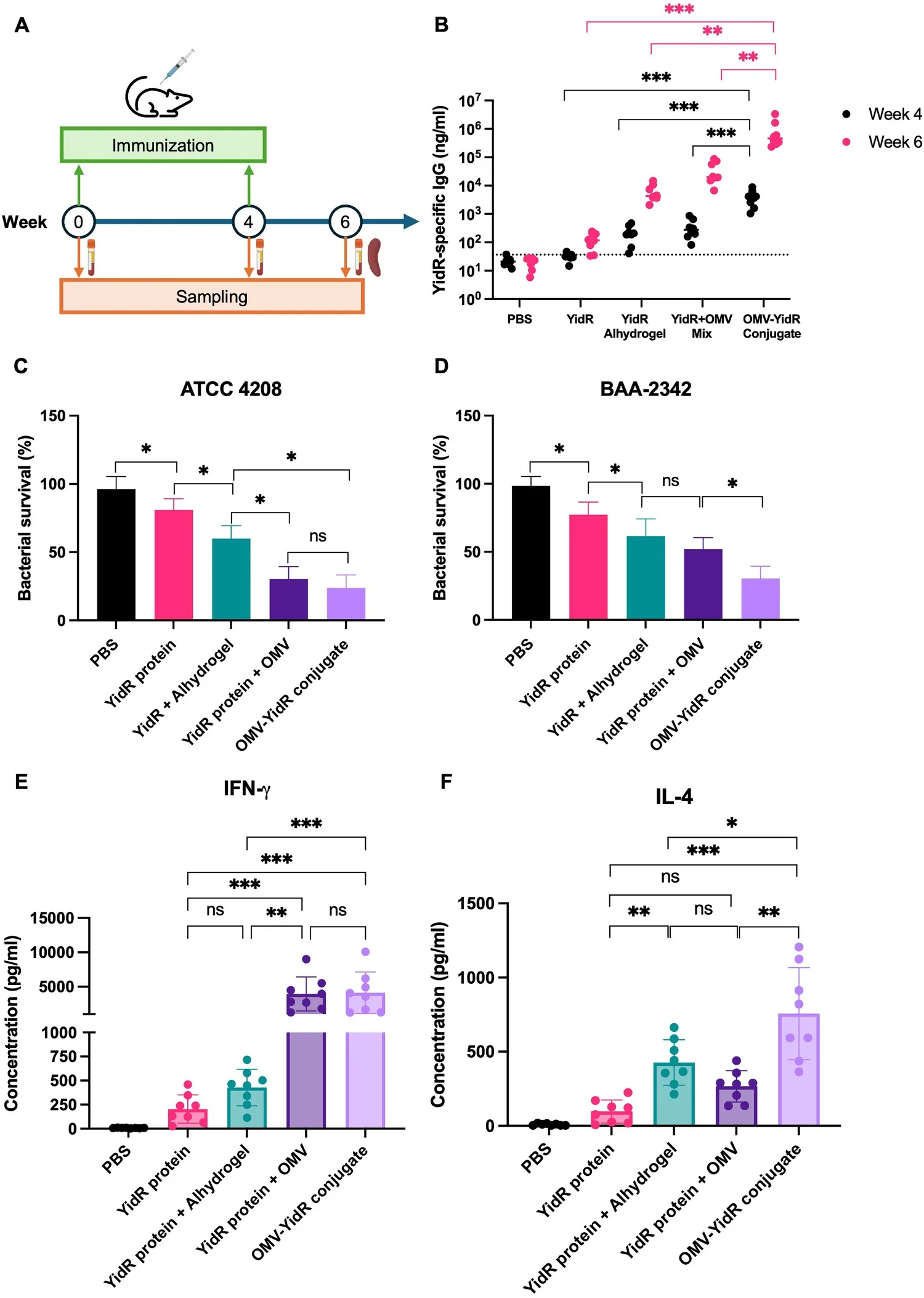
OMV-YidR conjugate offers more robust immune induction than physical mix of the two. (A) Immunization strategy. C57BL/6J mice were first immunized twice in at 4-week intervals and then serum samples were collected at week 0, 4 and 6 for antibody measurement and spleens were harvested at week 6 for T-cell mediated response evaluation. (B) YidR-specific IgG in serum samples was measured by ELISA (n = 8). (C–D) The OPK activity of immunized sera at Week 6 against homologous ATCC 4208 and heterologous BAA-2342 strains. ns, statistically not significant; *, *p* < 0.05. (E–F) Splenocytes were isolated at week 6 and restimulated ex vivo with purified YidR protein, and secreted (E) IFN-γ and (F) IL-4 were quantified by ELISA (*n* = 8). *, *p* < 0.05; **, *p* < 0.01; ***, *p* < 0.001.

To assess the intrinsic adjuvant properties of the OMV carrier and determine the specific immunological benefit of covalent conjugation, the immunogenicity of the OMV-YidR conjugate was compared to a simple physical mixture of OMV and YidR (OMV + YidR). YidR formulated with Alhydrogel (aluminium hydroxide) was included as a positive reference control. C57BL/6J mice were immunized at 4-week intervals, and immune responses were assessed at Weeks 4 and 6 (Figure 4(A)).

Consistent with previous observations, unadjuvanted YidR protein elicited a negligible IgG response, with titres remaining very low at Week 6. In contrast, the addition of either OMVs (physical mixture) or Alhydrogel significantly potentiated the humoral response; both the YidR + OMV mixture and the YidR + Alhydrogel reference group generated robust and comparable YidR-specific IgG titres at Week 4, which were further boosted after the second dose (Figure 4(B)). This confirms that the OMV carrier possesses substantial adjuvant activity comparable to the commercial standard. However, the covalent OMV-YidR conjugate demonstrated superior immunogenicity, eliciting significantly higher IgG titres at both Week 4 and Week 6 compared to either the physical mixture or the Alhydrogel reference. This enhancement indicates that the physical linkage of antigen and carrier provides a distinct immunological advantage over simple co-administration.

To further evaluate the immunological superiority of covalent conjugation over simple co-administration, we compared the functional bactericidal capacity of sera from mice immunized with the OMV-YidR conjugate, the OMV + YidR physical mixture, and the YidR protein only and YidR + Alhydrogel reference groups using an OPK assay (Figure 4(C, D)). In alignment with our quantitative IgG data, unadjuvanted YidR protein induced only negligible neutrophil-mediated killing against both the homologous ATCC 4208 and the heterologous MDR BAA-2342 strains. While the addition of Alhydrogel significantly enhanced opsonophagocytic activity, the YidR + OMV physical mixture demonstrated even greater potency against the homologous ATCC 4208 strain (Figure 4(C)). This enhancement is likely attributable to the synergistic effect of antibodies targeting both the YidR protein and the OMV-derived surface antigens of the production strain. However, a critical limitation of the physical mixture was observed against the heterologous MDR strain BAA-2342, where its killing activity was only comparable to the YidR + Alhydrogel group (Figure 4(D)). This suggests that while OMVs can serve as a potent adjuvant to boost anti-YidR responses, the antibodies generated against the ATCC 4208 OMV carrier lack broad cross-reactivity. Remarkably, the OMV-YidR conjugate elicited the highest level of functional bactericidal activity against both strains, significantly outperforming the physical mixture and the standard adjuvant group. These results provide direct mechanistic evidence that the covalent linkage of the antigen to the OMV surface is essential for maximizing the quality of the humoral response. This stable architecture not only exploits the OMV's intrinsic adjuvanticity but also ensures that the conserved YidR epitopes are presented in a manner that generates the broad-spectrum, high-titer functional antibodies required to empower neutrophils for multi-drug-resistant bacterial clearance.

To evaluate the impact of formulation on T cell response, splenocytes were harvested at Week 6 and restimulated *ex vivo* with purified YidR. The production of IFN-γ (Th1) and IL-4 (Th2) was quantified to profile the immune bias (Figure 4(E, F)). YidR protein alone induced very low levels of both cytokines. The Alhydrogel reference group showed increased levels of both cytokines but exhibited a distinct Th2 skew, with a more profound elevation in IL-4. In comparison, the YidR + OMV physical mixture also enhanced both cytokines but favoured a Th1 profile, inducing significantly higher levels of IFN-γ than the Alhydrogel group. When comparing the physical mixture to the conjugate, the OMV-YidR conjugate maintained the strong IFN-γ production seen in the mixture group, preserving the critical Th1 component. However, the conjugate formulation significantly improved IL-4 production compared to the physical mixture. These data suggest that while the OMV carrier naturally drives a Th1-dominant response, conjugating the antigen broadens the immune profile, which can boost the Th2 arm of Th1-biased immunity.

**Figure 5.**
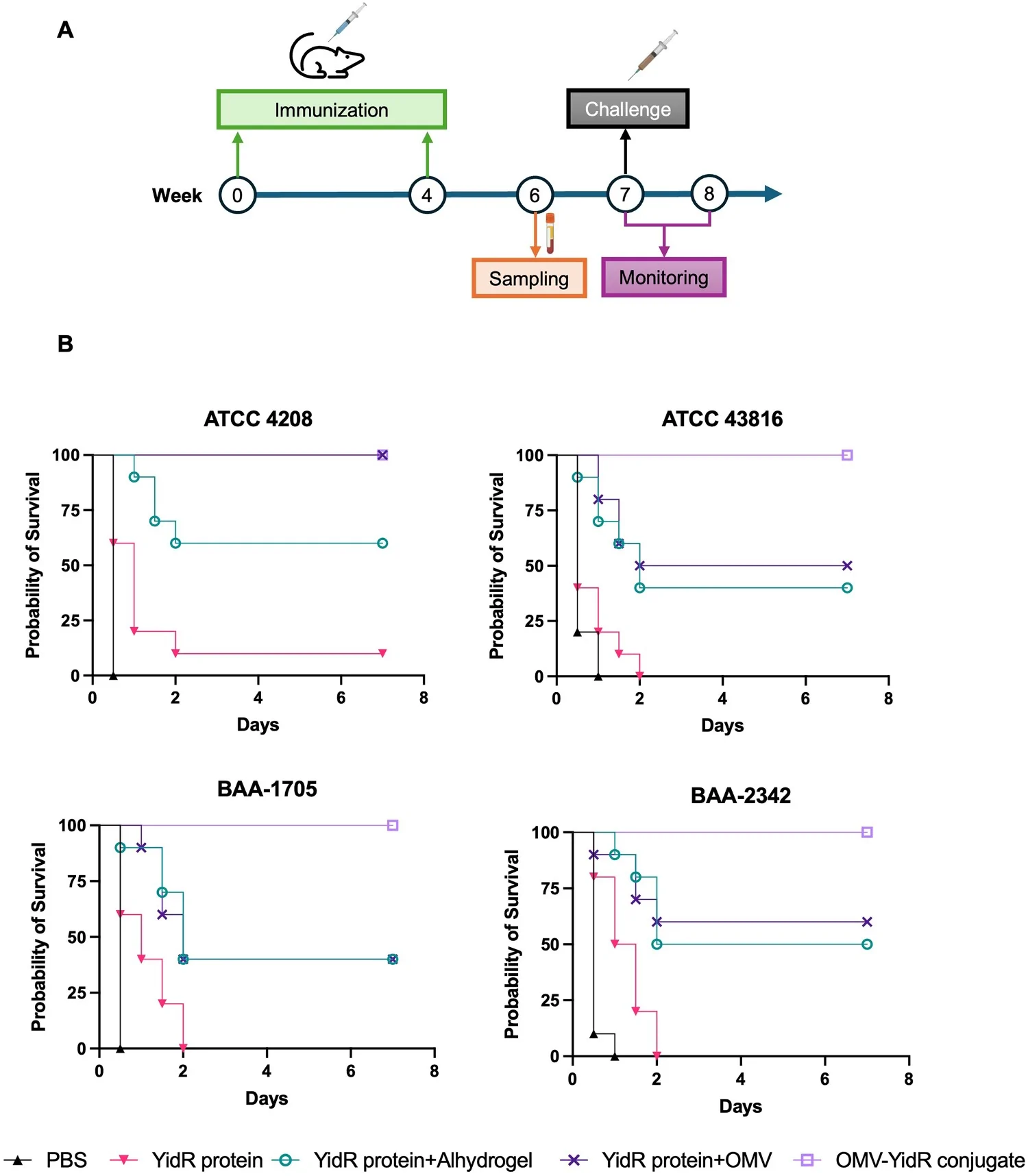
OMV-YidR conjugate induces protective immunity against homologous and heterologous KP challenge. (A) immunization and challenge strategy. C57BL/6J mice were first immunized twice at 4-week intervals and then challenged at week 7 with a lethal dose of homologous (ATCC 4208: 5 × 10^7 CFU) and heterologous KP strains (ATCC 43816: 5 × 10^4 CFU, BAA-1705: 1 × 10^8 CFU, and BAA-2342: 1 × 10^8 CFU). (B) Animal survival was monitored for 7 days after challenge (*n* = 10).

To evaluate the protective efficacy of the different vaccine formulations, a challenge study was performed against the same 4 distinct KP strains (Figure 5(A)). Our data showed that against the homologous ATCC 4208 strain, both the OMV-YidR conjugate and the OMV + YidR physical mixture provided complete (100%) survival. This indicates that against the parent strain, OMV-derived antigens provide a high level of protection. In contrast, the YidR + Alhydrogel group conferred only partial protection (60% survival), while soluble YidR alone offered negligible defence (10%) (Figure 5(B)).

The advantage of covalent conjugation became even more pronounced during challenges with heterologous MDR and hypervirulent strains. Against the hypervirulent strain ATCC 43816, the OMV-YidR conjugate maintained 100% survival, significantly outperforming the physical mixture (50% survival) and the Alhydrogel group (40% survival). Similar results were observed against the carbapenem-resistant isolates BAA-1705 and BAA-2342, where the conjugate provided 100% protection, while the physical mixture and Alhydrogel formulations achieved only 40–60% survival (Figure 5(B)).

These results demonstrate that while standard adjuvants and simple co-administration can enhance survival to a moderate degree, covalent linkage to the OMV carrier achieves enhanced, broad-spectrum protective immunity which survives challenges with highly virulent and multi-drug resistant KP lineages.

**Figure 6.**
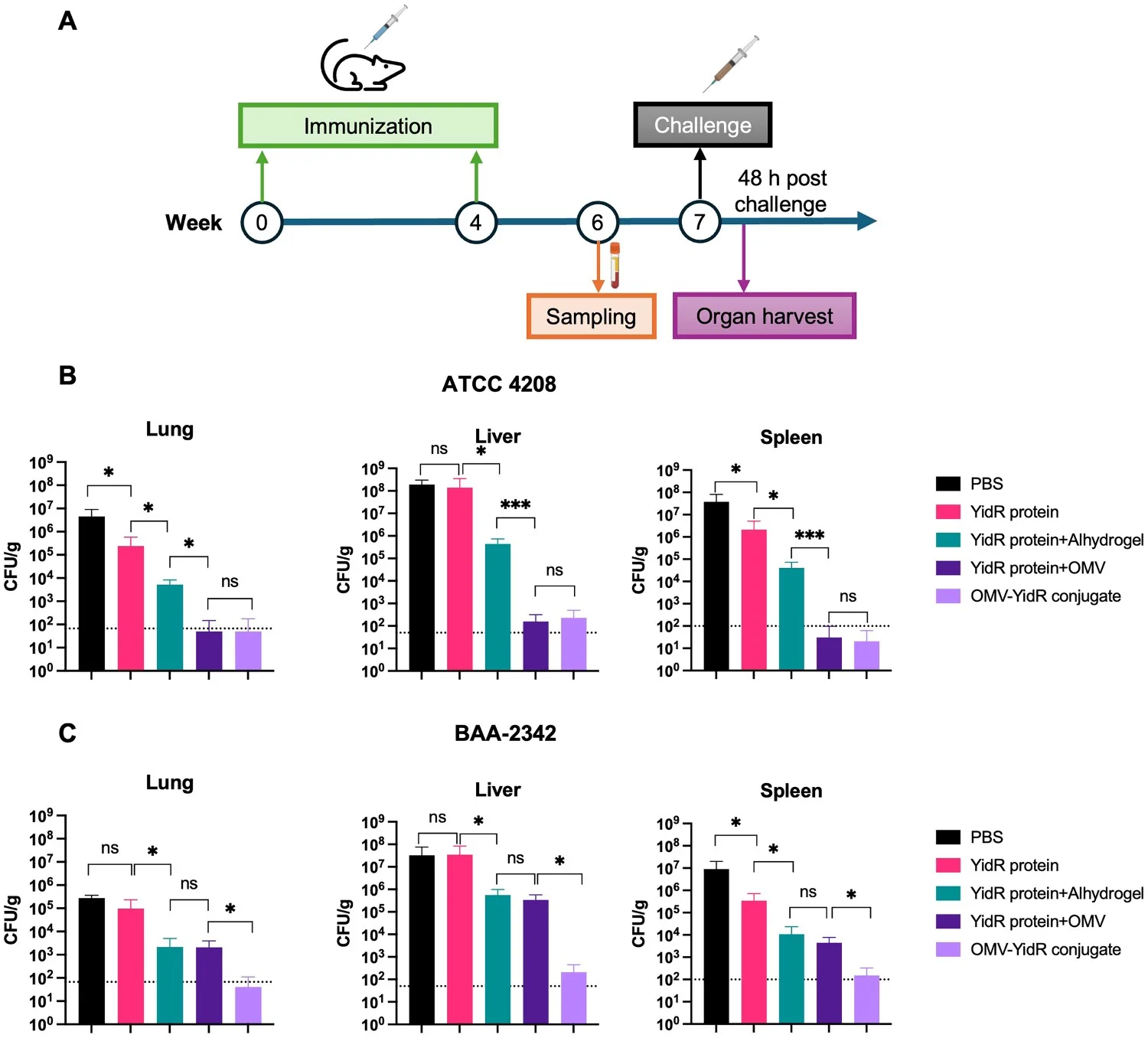
OMV-YidR conjugate vaccination prevents systemic dissemination and facilitates multi-organ bacterial clearance. (A) Experimental timeline for intramuscular immunization, non-lethal i.p. challenge, and organ harvest at 48 h post-challenge. (B–C) Viable bacterial loads in the lung, liver, and spleen of immunized mice 48 h after challenge with (B) homologous ATCC 4208 or (C) heterologous MDR BAA-2342 strains (*n* = 5 per group). Dotted horizontal lines represent the limit of detection. *, *p* < 0.05; ***, *p* < 0.001; ns, non-significant.

To address the impact of vaccination on systemic pathogen dissemination, tissue bacterial burdens were evaluated in the lungs, liver, and spleen of vaccinated mice 48 h after challenge with non-lethal doses of either the homologous ATCC 4208 or heterologous BAA-2342 strains (Figure 6(A)). Following challenge with homologous ATCC 4208, unadjuvanted YidR protein failed to induce significant clearance in the liver compared to the PBS control, though it mediated minor, statistically significant reductions in the lung and spleen (Figure 6(B)). Adjuvantation with Alhydrogel improved bacteria clearance in all 3 organs compared to the unadjuvanted YidR protein control (Figure 6(B)). Notably, both OMV-containing formulations – the YidR protein + OMV mixture and the OMV-YidR conjugate – demonstrated superior protective clearance against the homologous strain, reducing the liver bacterial burden to near the limit of detection and driving the lung and spleen burdens below detectable levels (Figure 6(B)). No statistically significant difference in clearance was observed between the physical mixture and the covalent conjugate against this homologous parent strain.

The challenge with the heterologous MDR BAA-2342 strain revealed distinct efficacy profiles between the YidR protein + OMV mixture and the OMV-YidR conjugate vaccine (Figure 6(C)). While unadjuvanted YidR protein again showed minimal clearance benefit, both the YidR + Alhydrogel and YidR + OMV mixture groups yielded moderate, comparable reductions across all three target organs (Figure 6(C)). Unlike the homologous ATCC 4208 challenge, the YidR + OMV mixture failed to achieve sterilizing clearance in the liver and spleen, reflecting the strain-specific limitations of anti-OMV antibodies. In contrast, the covalently linked OMV-YidR conjugate significantly outperformed all other groups (Figure 6(C)). The conjugate vaccine successfully drove the bacterial burden in the liver and spleen down to the limit of detection, and was the only formulation capable of completely clearing the bacterial load in the lungs to undetectable levels (Figure 6(C)). These findings confirm the benefits of covalent conjugation in eliciting the high-quality, broadly cross-reactive immune responses against heterologous KP strains.

### Covalent linkage to OMVs drives preferential B-cell targeting and uptake in the draining lymph nodes

The data presented above demonstrate that the OMV-YidR conjugate elicits superior immune responses and protective efficacy compared to both the soluble YidR protein and the YidR + OMV physical mixture. To investigate the potential mechanisms driving this discrepancy, we evaluated how the distinct physical properties of the formulations – specifically, the larger particulate size of the OMV carrier versus the soluble monomer – influence antigen trafficking and cellular uptake within the dLNs. Because the dLNs serve as the primary command centre for initiating adaptive immunity, the efficiency of antigen delivery to this compartment and the specific localized cell types mediating uptake are critical determinants of the subsequent immune response [22, 23].

**Figure 7.**
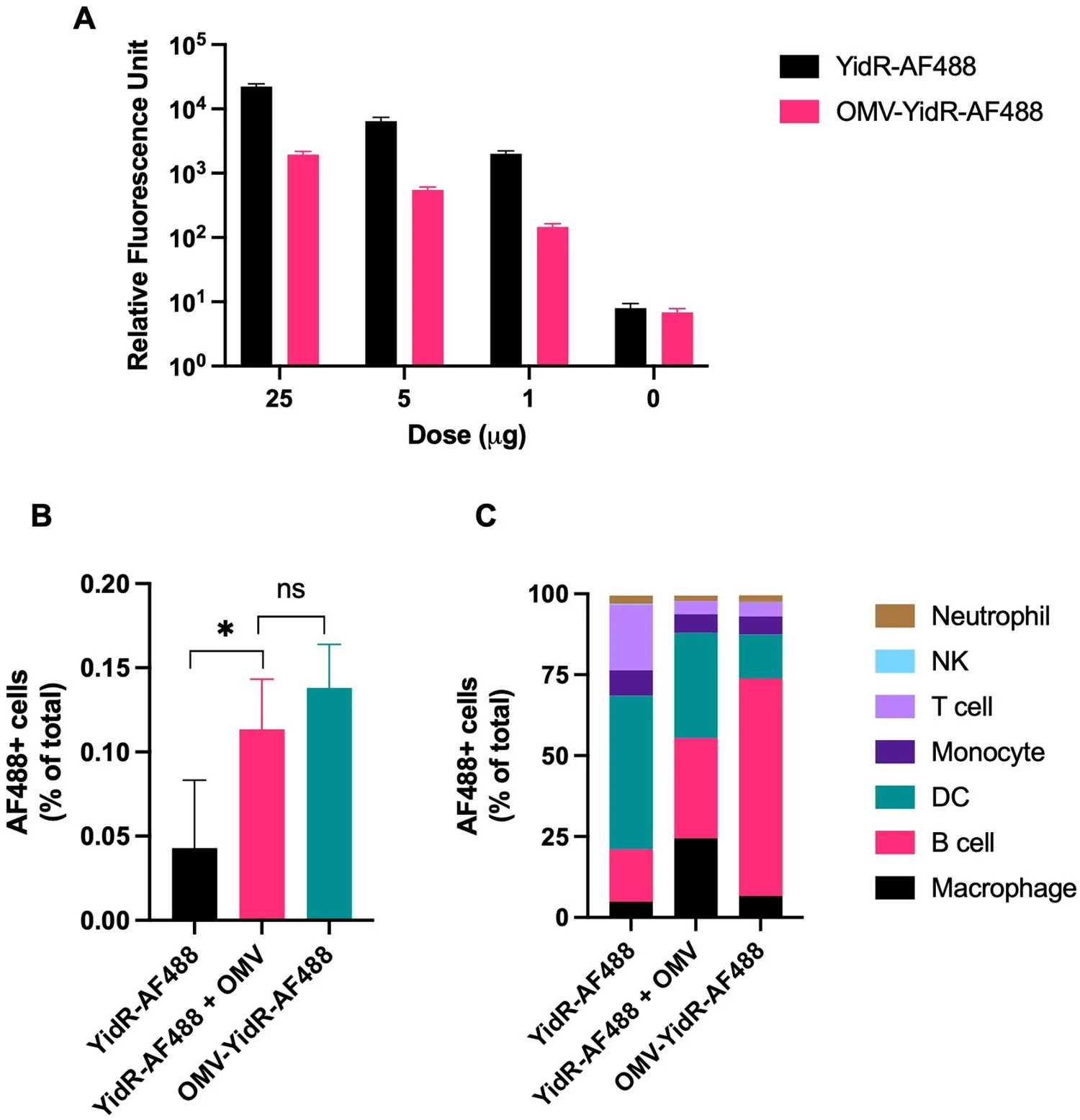
OMV conjugation alters antigen uptake and cellular biodistribution in dLN. (A) Fluorescent intensities of AF488-labelled YidR and OMV-YidR were measured in vitro at various doses. (B–C) C57BL/6J mice were first injected i.m. with YidR-AF488 alone, YidR + OMV physical mix or OMV-YidR-AF488, and then 1 h later, dLNs were isolated and (B) total AF488-positive cells and (C) AF488-positive neutrophils, NK cells, T cells, monocytes, DCs, B cells and macrophages were determined by flow cytometry (*n* = 5). ns, non-significant; *, *p* < 0.05.

To visualize this, purified YidR was labelled with AF488 and administered intramuscularly to mice either as soluble YidR-AF488, or soluble YidR-AF488 mixed with OMV, or conjugated OMV-YidR-AF488. The dLNs were harvested 1 h post-injection to quantify early cellular uptake. Prior to *in vivo* administration, the fluorescence signals of soluble YidR-AF488 and OMV-YidR-AF488 conjugate were characterized *in vitro*. At equivalent total protein concentrations, the soluble YidR-AF488 exhibited higher fluorescence intensity compared to the OMV-YidR-AF488 conjugate across the tested range (Figure 7(A)). Despite the lower specific fluorescence of the conjugate *in vitro*, analysis of the dLNs revealed a significantly higher accumulation of the antigen in the OMV-treated animals. The total number of AF488-positive cells was significantly elevated in the YidR-AF488 + OMV physical mix group and OMV-YidR-AF488 conjugate group compared to the soluble YidR-AF488 group (Figure 7(B)), indicating that the addition of OMV, either through simple mix or conjugation, facilitates more efficient transport to, or retention within, the lymph node environment and promotes antigen uptake. Total antigen accumulation in the conjugate group was marginally higher than in the physical mix group, though the difference did not reach statistical significance.

Furthermore, phenotypic analysis of the AF488-positive population revealed a distinct formulation-dependent shift in cellular tropism (Figure 7(C)). The uptake of soluble YidR was predominantly restricted to Dendritic Cells (DCs). When mixed with OMV, antigen uptake was broadly distributed across DCs, B cells, and an expanded macrophage population, suggesting that a substantial fraction of the co-administered free antigen is cleared non-specifically by professional phagocytes under a non-specific inflammatory bystander effect. In contrast, the OMV-YidR conjugate showed a B cell-dominant uptake profile (Figure 7(C)). This suggests that the OMV conjugation platform may promote enhanced antigen accumulation within the B cell compartment, potentiating better immune responses.

In conclusion, this study demonstrates that covalently conjugating the conserved YidR antigen to KP-derived OMVs successfully overcomes the protein's inherent weak immunogenicity to elicit potent, broad-spectrum immunity. By facilitating efficient antigen accumulation in B cells within the dLNs and driving a protective Th1/Th17 cellular bias, the conjugate formulation generated robust immune responses that soluble antigen or physical mixtures failed to achieve. This potentiation of immunity against a conserved target ultimately conferred complete cross-protection against diverse clinical isolates, including hypervirulent and carbapenem-resistant strains, positioning the OMV-YidR conjugate as a promising vaccine candidate for combating multi-drug resistant infections.

## Discussion

The escalating convergence of MDR and hypervirulence in KP underscores the urgent need for interventions that transcend the limitations of current antibiotic therapies. While vaccination represents an effective strategy to curb the spread of antimicrobial resistance (AMR), the development of a “universal” KP vaccine has been impeded by the immense diversity of capsular serotypes. In this study, we developed a novel nanovaccine by covalently conjugating the highly conserved YidR antigen to KP-derived OMVs. Our findings demonstrate that this conjugation strategy not only overcomes the weak intrinsic immunogenicity of soluble YidR but also broadens the protective range of OMV-based immunization, conferring 100% protection against lethal challenges with diverse hypervirulent and CRKP clinical isolates.

The choice of YidR as the target antigen was predicated on its high conservation (>97% homology) across diverse KP strains [10]. Previous attempts to utilize YidR as a vaccine candidate, including recombinant protein and DNA platforms, have shown promise in murine sepsis models [9, 11]. However, recombinant subunit vaccines generally require potent adjuvants to elicit durable immunity. Our data confirm that soluble YidR without adjuvant elicits negligible IgG titres and poor protection against homologous challenge. By contrast, the OMV-YidR conjugate elicited IgG titres over 20,000-fold higher than soluble protein. This dramatic enhancement suggests that the OMV serves not merely as a passive carrier but as a potent, intrinsic adjuvant, likely driven by the presence of pathogen-associated molecular patterns (PAMPs) such as LPS and outer membrane proteins that activate host pattern recognition receptors [24, 25]. Importantly, our comparison with physical mixtures revealed that co-delivery is not as efficient as physical linkage. The OMV-YidR conjugate elicited significantly higher IgG titres than the physical mixture, indicating that conjugation of antigen onto OMV may promote antigen processing and presentation.

A pivotal mechanistic insight from our study is the distinct alteration in cellular uptake profiles observed within the dLNs. While soluble YidR was primarily internalized by DCs, the OMV-YidR conjugate exhibited a preferential accumulation in B cells. This B cell-dominant uptake likely facilitates more efficient BCR cross-linking and antigen presentation, driving the robust seroconversion observed [26]. Furthermore, while traditional alum adjuvants skew responses towards Th2, OMV-based formulation drives a more Th1/Th17-biased response [27]. The induction of IL-17A is particularly significant, as Th17-mediated immunity is critical for mucosal defence against KP pulmonary infections [28]. This suggests that the chemical conjugation of antigen to the OMV surface physically links the “danger signal” of the OMV to the target antigen, thereby broadening the immune polarization to recruit both cellular and humoral arms of the adaptive immune system.

The limitations of OMV vaccines have historically been their strain-specific protection, often restricted by the immense variability of surface O-antigens and capsular polysaccharides (CPS) [4, 7, 8, 29]. Indeed, in our study, mice immunized with unconjugated OMVs (derived from ATCC 4208) succumbed rapidly when challenged with heterologous strains such as the hypervirulent ATCC 43816 or the resistant BAA-2342. However, the OMV-YidR conjugate conferred complete protection against these heterologous strains. This indicates that the conserved YidR epitopes, when displayed at high density on the OMV surface, can bypass the serotype-specific limitations of the OMV carrier itself. In addition, while ATCC 4208 was utilized as our primary OMV donor strain in this study, our pilot evaluations comparing OMVs from ATCC 4208 and BAA-2342 showed no significant differences in their capacity to adjuvant anti-YidR humoral responses (Figure S3). This “Plug-and-Display” versatility aligns with recent advancements in OMV engineering, such as the SpyCatcher/SpyTag systems used to display tumour antigens or S. aureus proteins, but utilizes a stable chemical conjugation approach that may be more translatable for large-scale manufacturing and regulatory approval [16, 17].

We acknowledge that while the OMV-YidR conjugate exhibits preferential accumulation in dLN B cells early after administration, isolating the direct causative role of this initial trafficking step from downstream effector functions remains an experimental challenge. Because B cells are required both for early follicular antigen capture/presentation and downstream antigen-specific IgG production, classical systemic B-cell depletion models would confound results by completely abolishing the humoral effector arm. Nonetheless, our head-to-head comparison of the covalent conjugate against the physical mixture provides compelling evidence of this link. Unconjugated physical mixing – which fails to redirect antigen trafficking to follicle B cells – is unable to trigger the same magnitude of IgG production or Th1/Th17 bias. Thus, while B-cell-dominant uptake is a key correlate of early particulate delivery, the final protective outcome is driven by a coordinated cascade where early follicular antigen-channeling optimizes downstream germinal centre selection and long-term humoral memory.

Despite these promising results, several limitations must be addressed. First, while we utilized KP-derived OMVs, the presence of LPS raises concerns regarding endotoxicity for human applications [30]. Future iterations of this vaccine could utilize genetically detoxified strains (e.g. ΔmsbB mutants) to attenuate LPS reactogenicity while maintaining adjuvanticity [31, 32]. Second, our study employed intramuscular immunization. Given that KP can be transmitted through the respiratory system, future studies could evaluate intranasal delivery of the OMV-YidR conjugate to determine if it can elicit superior mucosal responses and prevent colonization [33]. Third, this study evaluated vaccine immunogenicity and protective efficacy exclusively in female mice. While female cohorts were usually selected for early-stage proof-of-concept vaccine studies, future translational development, safety, and toxicology evaluations of the OMV-YidR platform should include both male and female cohorts to ensure the universal applicability of these findings [34, 35]. Lastly, we acknowledge that this study did not encompass a head-to-head comparative screening of YidR against other recently characterized conserved antigens, such as TamA, FimA, or LysM/BON [8, 36, 37]. Our primary objective was to establish a mechanistic and therapeutic proof-of-concept for the covalent OMV-conjugation platform. YidR served as a highly robust, universally conserved model antigen that allowed us to demonstrate that stable physical linkage – rather than simple physical mixing – is structurally required to alter cellular tropism and selectively channel antigens to the B-cell compartment in draining lymph nodes. Having successfully validated this platform-mediated enhancement, future translational work will focus on expanding this click-chemistry conjugation strategy to other conserved outer membrane assemblies and multi-antigen cocktails to further optimize the protective breadth of our nanovaccine platform.

In conclusion, our study establishes the OMV-YidR conjugate as a highly efficacious, mechanism-driven vaccine candidate. By combining the broad cross-reactivity of a conserved protein antigen with the potent, self-adjuvanting properties of an OMV carrier, this vaccine strategy bypasses traditional serotype limitations, offering a scalable and robust prophylactic solution to the escalating global crisis of multidrug-resistant *K. pneumoniae*.

## Supplementary Material

Supplementary_figures_.docx

## Data Availability

The raw data supporting the conclusions of this manuscript are available from the corresponding author, without undue reservation, to any qualified researcher upon reasonable request.
