# Supplementary material for: A universally conserved OMV-YidR conjugate nanovaccine confers broad protection against hypervirulent and carbapenem-resistant Klebsiella pneumoniae
Source: Emerg Microbes Infect. 2026 Sep 7;15(1):2731500. doi: 10.1080/22221751.2026.2731500 (PMC13587577; doi:10.1080/22221751.2026.2731500)
Supplement: Supplementary file 1 [file TEMI_A_2731500_SM4887.docx]

**Figure S1 (part 1).** OMV-YidR conjugate elicits enhanced cellular responses against YidR. C57BL/6J mice were first immunized twice in at 4-week intervals and then spleens were harvested at week 6 and splenocytes were isolated at week 6 and restimulated ex vivo with purified YidR protein, and secreted Th1/2/9/17/22/Treg-related cytokines were measured by Luminex (n = 8). Heat map shows the fold change of individual cytokine expression relating to control group (PBS group).

**Figure S1 (part 2).** OMV-YidR conjugate elicits enhanced cellular responses against YidR. C57BL/6J mice were first immunized twice in at 4-week intervals and then spleens were harvested at week 6 and splenocytes were isolated at week 6 and restimulated ex vivo with purified YidR protein, and secreted Th1/2/9/17/22/Treg-related cytokines were measured by Luminex (n = 8). Heat map shows the fold change of individual cytokine expression relating to control group (PBS group).

**Figure S2.** Identification of lethal dose for each KP strain. Naïve C57BL/6J mice were challenged with ascendant doses of KP strains (ATCC 4208, ATCC 43816, BAA-1705 and BAA-2342) through i.p. and animal survival was monitored for 7 days. The lowest dose that resulted in 100% animal death was chosen as the lethal dose for each strain.

**Figure S3.** OMV-YidR conjugate elicits enhanced humoral responses against YidR. C57BL/6J mice were first immunized twice in at 4-week intervals and then serum samples were collected at week 6 for YidR-specific IgG measurement by ELISA (n = 5). OMV4208: OMVs isolated from ATCC 4208 strain; OMV2342: OMVs isolated from BAA-2342 strain.
